# Non-invasive Evaluation of Brain Death Caused by Traumatic Brain Injury by Ultrasound Imaging

**DOI:** 10.3389/fninf.2020.607365

**Published:** 2020-11-16

**Authors:** Ningning Niu, Ying Tang, Xiaoye Hao, Jing Wang

**Affiliations:** Department of Ultrasound, Tianjin First Center Hospital, Tianjin, China

**Keywords:** brain death, ultrasound, echocardiography, intracranial pressure, transcranial Doppler

## Abstract

**Objectives:**

To investigate the clinical value of non-invasive ultrasound imaging in the evaluation of brain death caused by traumatic brain injury.

**Methods:**

Thirty-four patients with acute severe traumatic brain injury were admitted to hospital within 48 h after injury. All patients were monitored intracranial pressure, transcranial Doppler, echocardiography examination, collection intracranial pressure, MCA-Vs, MCA-Vd, MCA-Vm, EF, LVMPI, RVMPI and other indicators, and combined with clinical conditions and other related data for comparative study and statistical analysis.

**Results:**

The blood flow spectrum was characterized by diastolic retrograde blood flow spectrum pattern and nail waveform spectrum shape when the patient had clinical brain death. For the parameters of transcranial Doppler, there were significant differences in MCA-Vm and PI between clinical brain death group and normal control group (*P* < 0.05). For the parameters of echocardiography, there were statistically significant differences in EF, LVMPI, and RVMPI between clinical brain death group and normal control group (*P* < 0.05).

**Conclusion:**

Non-invasive dynamic monitoring of cerebral hemodynamics and cardiac function parameters in patients with severe craniocerebral injury can provide a high accuracy and reliability for the preliminary diagnosis of brain death in patients with severe craniocerebral injury. It is helpful for early evaluation of prognosis and provides effective monitoring methods and guidance for clinical treatment.

## Introduction

With the rapid development of economic construction, urbanization and transportation, the incidence of traumatic brain injury increasing gradually and the number of patients who died due to craniocerebral trauma are also increasing gradually. However, the mortality rate of patients with severe traumatic brain injury is extremely high. In clinical work, we found that many patients with severe traumatic brain injury have been in the state of spontaneous breathing arrest, deep coma, dilation and fixation of double pupil due to intracranial hypertension or other reasons, and they are completely dependent on the ventilator to maintain breathing, that is, the brain function has been completely irreversible damage. In this case, the maintenance of respiration and drug circulation by ventilator often can maintain the heart rate and circulation for a long time until the organs of the whole body fail. This not only wastes valuable medical resources, but also limits the development of organ transplantation. Brain death will produce a series of pathophysiological changes including hemodynamics, endocrine, metabolism, inflammatory reaction, among which the most prominent manifestation is hemodynamic disorder. Non-invasive dynamic monitoring of cerebral hemodynamics and cardiac function parameters in patients with severe traumatic brain injury by ultrasound imaging is conducive to early evaluation of the prognosis of patients, providing effective monitoring means and guiding methods for clinical treatment, so as to provide a favorable opportunity for organ transplantation.

## Materials and Methods

### Subjects

**FIGURE 1:**
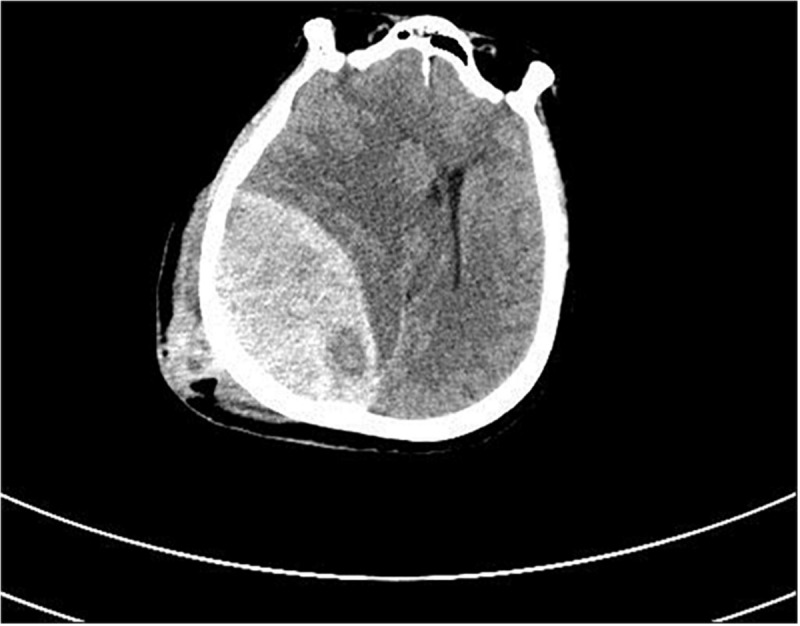
The CT examination of one patient with cerebral contusion and laceration with intracerebral hematoma.

Thirty-four patients with acute severe traumatic brain injury were admitted to hospital within 48 hours after injury. There were 30 males and 4 females, aged 26–53 years old. The injury mechanism included 17 cases of accelerated injury, 13 cases of deceleration injury and 4 cases of mixed injury. Among them, 18 cases were injured by traffic accidents, 8 cases were caused by falling, 5 cases were injured by striking, and 3 cases were injured by other reasons. All cases were diagnosed by CT examination before or after admission. According to CT diagnosis, brain injury was classified. The main injury types included: 15 cases of cerebral contusion and laceration combined with acute subdural hematoma, 11 cases of cerebral contusion and laceration with intracerebral hematoma, 6 cases of acute epidural hematoma, and 2 cases of extensive brain contusion and brain swelling. According to the state of consciousness, 19 patients with GCS score of 6-8 and 15 patients with GCS score less than 6. Among them, 9 patients with multiple injuries, 5 patients with closed fractures of extremities (early manual reduction and plaster fixation in orthopedics department, and then open reduction and internal fixation according to the condition of neurosurgery) and 4 patients with pulmonary contusion were treated conservatively (Figure 1).

All the patients were monitored intracranial pressure and dynamic cerebral hemodynamic indexes of transcranial Doppler after admission. According to the prognosis of the patients, they were divided into clinical brain death group (see the following clinical brain death diagnostic criteria) and survival group. Another 20 healthy subjects were selected as the control group, and laboratory, transcranial Doppler and echocardiography were performed respectively. All measurements were performed by a physician with 5 years of experience in ultrasonography.

### Inclusive Criteria and Exclusion Criteria

*Inclusive criteria*: Admission within 48 h after injury; age 20–60 years old; GCS ≤ 8 points at admission.

*Exclusion criteria*: Patients with severe multiple trauma, combined injury and shock; those who have been transferred to our hospital after craniotomy in another hospital; patients with heart, lung, liver, and kidney dysfunction; patients with stenosis of internal carotid artery system and vertebrobasilar artery system; patients whose temporal window was completely closed and could not detect blood flow signals; patients whose family members gave up treatment.

### Clinical Diagnostic Criteria for Brain Death

The patients were clinically diagnosed as brain death. The criteria for brain death were referred to the criteria for brain death (adult) (Revised Version) drafted by the drafting group of the Ministry of Health in 2003 and the criteria and technical specifications for brain death (adult quality control version) in 2013. (i) Deep shock; (ii) disappearance of the papillary light reflex and corneal reflection; (iii) absence of spontaneous respiration; (iv) electroencephalogram displaying resting potential; (v) negative atropine test; (vi) no change in all of these conditions for 12 h after initial observation, then brain death was determined (Su et al., 2018).

### Intracranial Pressure Detection Monitoring

Intracranial pressure detection monitoring was carried out by intracranial pressure monitor (Canimo MPM-1, United States), with an intracerebral probe, and the patients who needed outdoor drainage used intraventricular probe. ICP monitoring lasted for 1 week, and some patients were slightly prolonged according to their needs. Head wounds should be cleaned and disinfected daily to prevent intracranial infection. If the baseline of intracranial pressure monitor is shifted due to the patient’s turning over or other nursing work, the baseline should be adjusted to zero according to the situation.

### Transcranial Doppler Examination

The cerebral hemodynamics was detected by transcranial Doppler (TCD) (DWL Multi-Dop X_2_, Germany). The middle cerebral artery was examined from the temporal window with a 2 MHz probe at a sampling depth of 45–60 mm. When the spectrum shape is smooth and the sound in the frequency window is loud was taken as the parameter index. All patients were placed in supine position with the head of bed raised 30 degrees. The detection time was 9:00 a.m. (before mannitol) and 4:00 p.m. (1 h after mannitol) for 7 days. After reaching the standard of clinical brain death, the patients were checked every 2–6 h according to their circulatory system. In order to exclude the influence of human subjective factors and technical factors on the experimental value, the cerebral blood flow detector was relatively fixed. The indexes included pulsatility index (PI), resistance index (RI), systolic blood flow velocity (MCA-Vs), diastolic blood flow velocity (MCA-Vd), and mean middle cerebral artery velocity (MCA-Vm).

### Echocardiography

Echocardiography was detected by ultrasound diagnostic system (Mindray M7, China) with a S4 probe at a frequency of 1–4 MHz. The patient was in supine position. The weight, height and blood pressure were recorded and BSA was calculated. The indexes of echocardiographic parameters included left ventricular end diastolic diameter (LVDd), left ventricular end systolic diameter (LVDs), and ejection fraction (EF). The myocardial performance index (MPI) was calculated by the ratio of the sum of isovolumic systolic (ICT) and isovolumic diastolic (IRT) to ejection time (ET), MPI = (ICT + IRT)/ET. The blood flow spectrum of mitral valve, tricuspid valve, aortic valve, and pulmonary valve was clearly displayed to calculate left ventricular (LVMPI) and right ventricular (RVMPI) according to the formula. In order to reduce the error of echocardiography as much as possible, the average value of three consecutive cardiac cycles was taken for each index. All examinations were completed by one doctor and the images were stored at the same time.

### Statistical Analysis

Statistical analysis was performed using SPSS Version 19.0 (IBM, Armonk, NY, United States). Continuous variables (normally distributed) were expressed as the mean ± standard deviation (SD). The differences between the two groups were compared by independent sample *t* test. The correlations were analyzed using Spearman’s correlation analysis, and the correlation coefficients (*r*) were calculated. A two-sided *P-*value < 0.05 was considered to indicate statistical significance.

## Results

### Changes of ICP and Cerebral Blood Flow Spectrum During Clinical Brain Death

**FIGURE 2:**
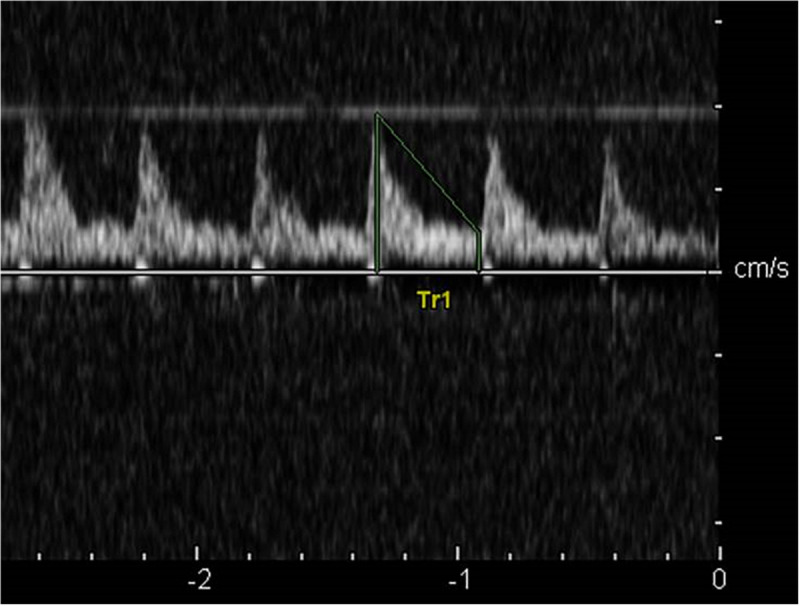
The cerebral blood flow spectrum in the control group.

**FIGURE 3:**
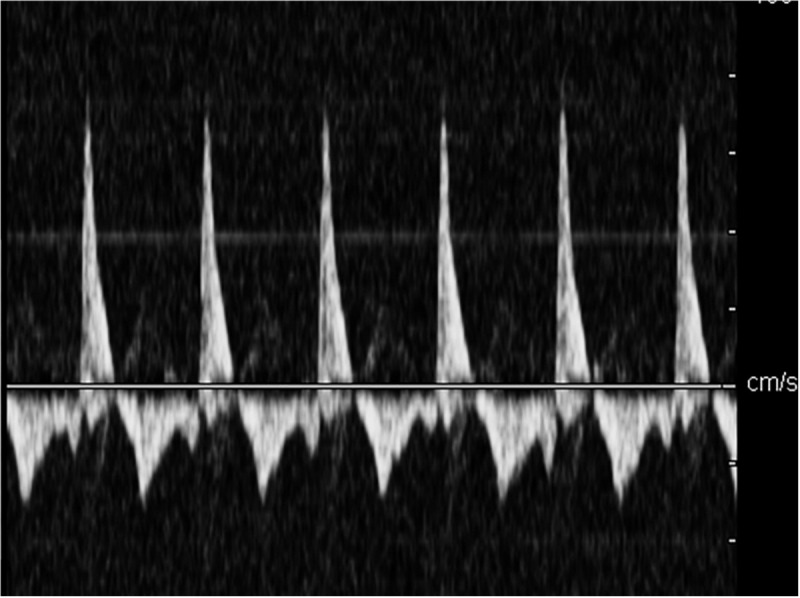
The diastolic retrograde blood flow spectrum pattern when patient had clinical brain death.

In the control group, the cerebral blood flow spectrum was characterized by high flow velocity and low resistance, the spectrum pattern was smooth, and the sound of blood flow signal was loud (Figure 2). In the acute stage of traumatic brain injury, the spectrum of cerebral blood flow in this group showed high peak in systolic phase, low and high in diastolic phase, which was the characteristic spectrum manifestation of increased intracranial pressure, ICP was positively correlated with PI (*r* = 0.872, *P* < 0.001) and MCA-Vm (*r* = 0.562, *P* = 0.003). With the increase of ICP, the amplitude of cerebral blood flow spectrum gradually decreased, and the MCA-Vm gradually decreased, showing a low flow rate and high resistance spectrum morphology. When the patient had clinical brain death, the blood flow spectrum was characterized by diastolic retrograde blood flow spectrum pattern and nail waveform spectrum shape (Figure 3). At this time, ICP of intracranial pressure monitoring was more than 70 mmHg, which exceeded the average arterial pressure of the patient. The ICP of some patients fluctuate at about 100 mmHg before clinical death.

### Comparisons of TCD Parameters

In this series of data, 15 patients with severe cerebral trauma has the characteristic performance of sum or nail spectrum by TDI before clinical brain death, and 14 patients were confirmed to have clinical brain death. The characteristic spectrum changes of cerebral perfusion arrest appeared earlier than clinical brain death. After the diagnosis of clinical brain death was established, the spectrum pattern of cerebral blood flow showed the spectrum pattern of nail spectrum or characteristic cerebral perfusion stop without blood flow signal. There were significant differences in MCA-Vm and PI between clinical brain death group and normal control group. The cerebral blood flow index was significantly lower after severe traumatic brain injury, than that of the normal control group, and PI was significantly higher than that of the normal control group (*P* < 0.05) (Table 1).

**TABLE 1:** Parameters of TCD in brain death group and normal group.

**Group**	***N***	**MCA-Vm**	**PI**	**ICP**
		**(cm/s)**		**(mmHg)**
Normal	20	58.55 ± 7.05	0.81 ± 0.16	
Brain death	14	15.82 ± 7.99	3.56 ± 1.13	68.18 ± 19.62
*t*		18.89	2.46	
*P*		0.001	0.001	

*Statistically significant difference compared with control group (*P*-value < 0.05). TCD, transcranial Doppler; MCA-Vm, mean middle cerebral artery velocity; PI, pulsatility index; ICP, intracranial pressure detection.*

### Comparisons of Echocardiography Parameters

**FIGURE 4:**
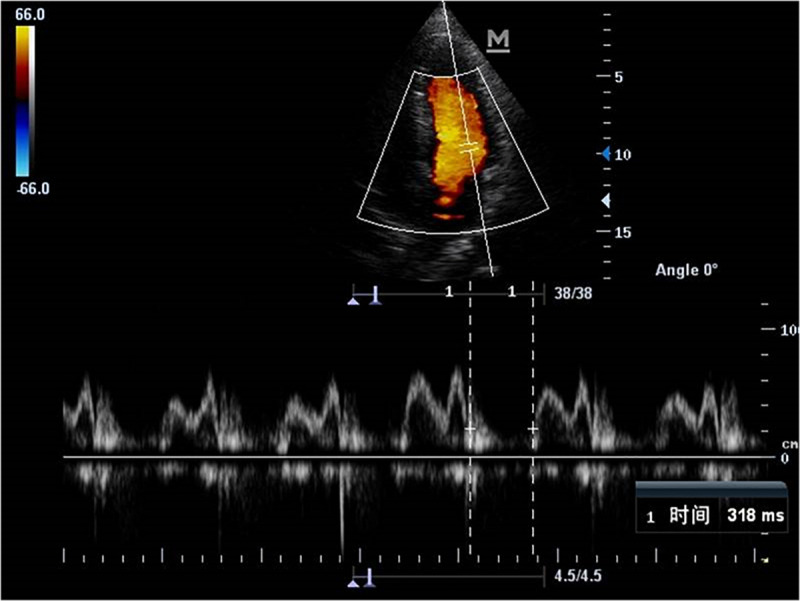
The blood flow spectrum of mitral valve in patient with clinical brain death.

**FIGURE 5:**
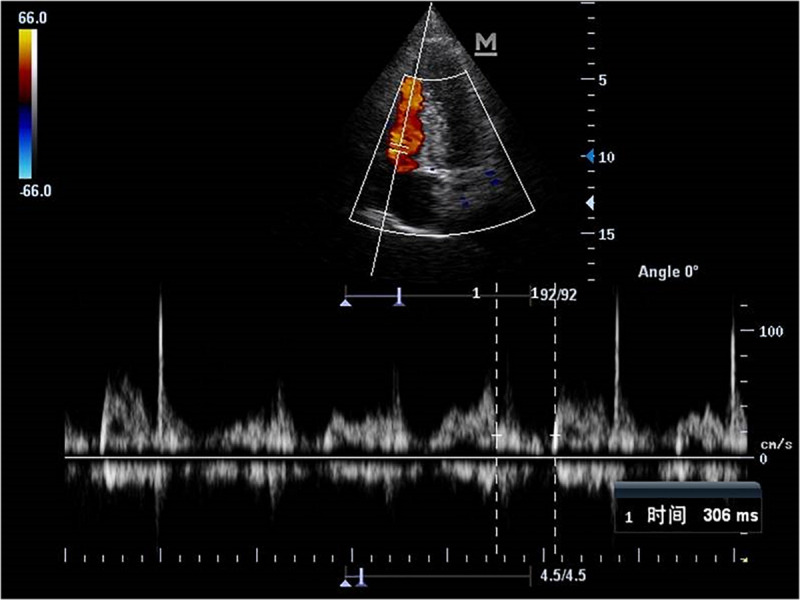
The blood flow spectrum of tricuspid valve in patient with clinical brain death.

Echocardiography parameters were performed in brain death group and normal control group (Figures 4–5). There were statistically significant differences in EF, LVMPI, and RVMPI between clinical brain death group and normal control group. LVMPI and RVMPI were higher in clinical brain death group than those in normal control group, and EF was lower than that in normal control group (*P* < 0.05) (Table 2).

**TABLE 2:** Parameters of echocardiography in brain death group and normal group.

**Group**	**LVDd**	**LVDs**	**EF**	**LVMPI**	**RVMPI**
	**(cm)**	**(cm)**			
Normal	45.61 ± 10.37	30.64 ± 10.15	61.57 ± 3.27	0.56 ± 0.13	0.25 ± 0.11
Brain death	46.33 ± 11.25	32.16 ± 10.82	59.38 ± 3.54	0.66 ± 0.16	0.41 ± 0.12
*t*	0.893	2.048	−2.667	1.507	3.902
*P*	0.377	0.084	0.01	0.05	0.008

*Statistically significant difference compared with control group (*P*-value < 0.05). LVDd, left ventricular end diastolic diameter; LVDs, left ventricular end systolic diameter; EF, ejection fraction; LVMPI, the myocardial performance index of left ventricular; RVMPI, the myocardial performance index of right ventricular.*

## Discussion

Severe craniocerebral trauma patients die because of the sharp increase of intracranial pressure and the formation of cerebral hernia, which is the difficulty of clinical treatment in neurosurgery. With the continuous development of Neurosurgery, the effect of rescue treatment has been improved, but the effect is not satisfactory (Shrestha et al., 2018). The maintenance of cerebral perfusion and cerebral circulation depends on reasonable intracranial pressure and mean arterial blood pressure. The cerebral hemodynamic state changes with the increase of intracranial pressure. The main pathophysiological manifestations are insufficient cerebral perfusion, ischemia and hypoxia of brain tissue, aggravation of brain edema, even cerebral circulation stop, resulting in clinical brain death.

Brain death refers to the irreversible stop of whole brain function due to persistent and severe cerebral ischemia, hypoxia or other reasons (Tang et al., 2019). The diagnosis of brain death requires comprehensive clinical conditions and a variety of examination methods (Mullaguri et al., 2018). At present, there are a variety of detection methods to help confirm brain death, including radionuclide scanning, cerebral angiography and so on. These examination methods have important value for the diagnosis of brain death. However, the instruments and equipment for these examinations are expensive, and most of them are invasive examinations, which need to be transported to specific examination rooms. There are many inconveniences in clinical application, which limit these methods as routine examination methods. Electroencephalogram (EEG) can continuously monitor patients with craniocerebral injury, but EEG detection is based on EEG resting as the diagnostic criteria of brain death, and there are still many problems in EEG resting as the diagnostic criteria, which has been controversial in clinical practice (Kasapoglu et al., 2019). In recent years, some scholars have found that not all patients with clinical brain death have EEG resting state, but some patients have extensive EEG activities.

Transcranial Doppler can monitor the cerebral hemodynamic status of patients with severe traumatic brain injury, describe the cerebral hemodynamic state combined with mean arterial blood pressure, and evaluate the cerebral perfusion and intracranial pressure (Cacciatori et al., 2018; Berthoud et al., 2019). Invasive intracranial pressure monitoring is the most direct and accurate detection method for patients with intracranial pressure, which is currently recognized as the gold standard for intracranial pressure detection (Roth et al., 2019). In this study, we continuously monitored the changes of intracranial pressure in patients with severe traumatic brain injury, combined with the monitoring of mean arterial blood pressure. At the same time, the relevant parameters of cerebral hemodynamics were dynamically detected by TCD.

The results showed that, with the slight increase of intracranial pressure, the condition was aggravated, MCA-Vd decreased in the early stage, while MCA-Vs increased slightly, and MCA-Vm was normal; with the moderate and severe increase of intracranial pressure, the condition was further aggravated, MCA-Vd and MCA-Vs decreased, MCA-Vd decreased more significantly, and PI increased gradually. When the blood pressure reaches the mean arterial blood pressure level and continues, diastolic regurgitation or nail spectrum may appear finally. The changes of parameters of cerebral blood flow state were significantly corresponding to the changes of intracranial pressure and disease condition. When patients with complete diastolic reverse blood flow or nail waveform, clinical brain death will occur in a short period of time. This indicates that the brain function of the patients has undergone serious and completely irreversible changes when the above-mentioned manifestations of cerebral blood flow state detected by non-invasive detection, indicating that the patient is about to or has suffered from clinical brain death.

Therefore, in the process of clinical treatment, before the typical spectrum of complete diastolic reverse blood flow or nail spectrum appears in TCD detection, active treatment should be taken as soon as possible to effectively control and reduce the improvement of cerebral perfusion and cerebral ischemia and hypoxia, which is of great significance for improving the prognosis and reducing the mortality of patients.

As an important organ to maintain hemodynamics, the heart can cause secondary heart injury in severe brain injury, especially in brain death (El-Battrawy et al., 2018). The lower the Lasco score is, the greater the decrease of EF would be. Brain death can lead to decreased cardiac systolic function, left ventricular ejection fraction, and ventricular wall motion.

As a non-invasive and rapid technique, ultrasound can dynamically monitor the structure and function of the heart. Myocardial performance index (MPI) as a comprehensive index to evaluate the overall systolic and diastolic function of the heart can be used to evaluate the overall ventricular function (Askin et al., 2018; Maria et al., 2019). When left ventricular systolic or diastolic dysfunction occurs, the isovolumic systolic period (ICT), isovolumic diastolic period (IRT), and ejection time (ET) change accordingly. Therefore, the measured value of MPI will also change accordingly, but it is not affected by many conditions such as age, heart rate, valve regurgitation, preload and afterload, cardiac geometry, two-dimensional image quality and angle between sampling line and blood flow direction. For patients with no change in left ventricular configuration, the change of global ventricular function can be evaluated only when diastolic function changes. In this study, the results showed that EF was lower in clinical brain death group than that in normal control group, LVESD, LVMPI, and RVMPI were higher than those in normal control group. There were significant differences in LVESD, EF, LVMPI, and RVMPI between clinical brain death group and normal control group.

Therefore, once the typical spectrum of complete diastolic reverse blood flow or nail spectrum appear in TCD detection, EF decreases and LVMPI, RVMPI increased in Echocardiography, that the patient has entered the state of brain death, and it is of no medical significance to continue treatment. With the informed consent of patients’ families, we should improve the systematic examination methods of brain death as soon as possible, such as EEG and brainstem evoked potential, to further diagnose brain death and save limited medical resources. Whether the patients who died of non-traumatic brain injury also have the characteristic blood flow changes of the above mentioned brain death needs further data collection and research. Non-invasive dynamic monitoring of cerebral hemodynamics and cardiac function parameters in patients with severe craniocerebral injury can provide a high accuracy and reliability for the preliminary diagnosis of brain death in patients with severe craniocerebral injury. It is helpful for early evaluation of prognosis and provides effective monitoring methods and guidance for clinical treatment. With the development of medical image data, it is helpful to further dig out the biometrics and other information in the image, which will further improve the accuracy of prediction (Zheng et al., 2020a,b).

## Data Availability Statement

The original contributions presented in the study are included in the article/supplementary material, further inquiries can be directed to the corresponding author.

## Ethics Statement

The studies involving human participants were reviewed and approved by the Medical Ethics Committee of the Tianjin First Central Hospital. The patients/participants provided their written informed consent to participate in this study.

## Author Contributions

YT contributed to the conception of the study and helped perform the analysis with constructive discussions. XH, NN, and JW performed the experiment. NN contributed to the data analysis and wrote the manuscript. All authors contributed to the article and approved the submitted version.

## Conflict of Interest

The authors declare that the research was conducted in the absence of any commercial or financial relationships that could be construed as a potential conflict of interest.
